# How much are we spending? The estimation of research expenditures on cardiovascular disease in Canada

**DOI:** 10.1186/1472-6963-12-281

**Published:** 2012-08-28

**Authors:** Claire de Oliveira, Van Hai Nguyen, Harindra C Wijeysundera, William W L Wong, Gloria Woo, Peter P Liu, Murray D Krahn

**Affiliations:** 1 University Health Network, Toronto, ON, Canada; 2 Toronto Health Economics and Technology Assessment Collaborative, Toronto, ON, Canada; 3 Faculty of Pharmacy, University of Toronto, Toronto, ON, Canada; 4 Schulich Heart Centre, Division of Cardiology, Department of Medicine, Sunnybrook Health Sciences Centre, University of Toronto, Toronto, ON, Canada; 5 Department of Medicine, University of Toronto, Toronto, ON, Canada

**Keywords:** Cardiovascular disease, Research expenditures, Health policy

## Abstract

**Background:**

Cardiovascular disease (CVD) is a leading cause of death in Canada and is a priority area for medical research. The research funding landscape in Canada has changed quite a bit over the last few decades, as have funding levels. Our objective was to estimate the magnitude of expenditures on CVD research for the public and charitable (not-for profit) sectors in Canada between 1975 and 2005.

**Methods:**

To estimate research expenditures for the public and charitable sectors, we compiled a complete list of granting agencies in Canada, contacted each agency and the Canadian Institutes of Health Research (CIHR), and extracted data from the organizations’ annual reports and the Reference Lists of health research in Canada. Two independent reviewers scanned all grant and fellowship/scholarship titles (and summary/key words, when available) of all research projects funded to determine their inclusion in our analysis; only grants and fellowships/scholarships that focused on heart and peripheral vascular diseases were selected.

**Results:**

Public/charitable sector funding increased 7.5 times, from close to $13 million (in constant dollars) in 1975 to almost $96 million (in constant dollars) in 2005 (base year). The Medical Research Council of Canada (MRCC)/CIHR and the Heart & Stroke Foundation of Canada have been the main founders of this type of research during our analysis period; the Alberta Heritage Foundation for Medical Research and the Fonds de la recherche en santé du Quebec have played major roles at the provincial level. The Indirect Costs Research Program and Canada Foundation for Innovation have played major roles in terms of funding in the last years of our analysis.

**Conclusion:**

Public/charitable-funded research expenditures devoted to CVD have increased substantially over the last three decades. By international standards, the evidence suggests Canada spends less on health-related research than the UK and the US, at least in absolute terms. However, this may not be too problematic as Canada is likely to free-ride from research undertaken elsewhere. Understanding these past trends in research funding may provide decision makers with important information for planning future research efforts. Future work in this area should include the use of our coding methods to obtain estimates of funded research for other diseases in Canada.

## Background

Cardiovascular diseases (CVD) are responsible for about one third of all deaths in Canada, and thus pose a considerable burden on society [1]. Not surprisingly, CVD is a priority focus for health research.

In Canada, public efforts to fund medical research date back to the first half of the 20^th^ century. In the early 1930s, the National Research Council and the Department of Agriculture were the main funding organizations, providing support for research related to human illness. In the late 1950s, the Medical Research Council of Canada (MRCC) was created to address the increasing need for additional research funding. It also served as an independent body that advised the government on policy and matters relating to medical research and its administration. From 1960 to 1990, the MRCC’s funding from the Government of Canada multiplied almost one-hundredfold, from $2.3 million to more than $200 million [2]. During that time, the MRCC was the major grant-funding agency for medical research in Canada [3]. The Canadian Institutes of Health Research (CIHR) replaced the MRCC in 2000 [4] and had a much broader mandate than its predecessor, supporting a wide spectrum of health research, from basic science to clinical research to health services and population health [5]. It was also endowed with a much larger budget — by its second year of operation, the annual budget of the CIHR was close to $500 million, nearly double that of the MRCC [5]. By 2005, the CIHR provided roughly 12 percent of all health research funding, making it the largest single funder in Canada [6].

Little has been published on how the magnitude and source of research expenditures have changed over time. Understanding past trends in research funding may provide decision makers with important information for planning future research efforts. For example, in 2008, the US National Institutes of Health (NIH) launched a major effort to code funds by disease area, the Research, Condition, and Disease Categorization (RCDC) [ http://report.nih.gov/rcdc/] to help obtain accurate estimates of funded research. Our analysis addresses this gap in knowledge by estimating the magnitude of past spending on CVD (heart and peripheral vascular disease) research from the non-profit (public/charitable) sector in Canada from 1975 to 2005.

## Methods

**Figure 1:**
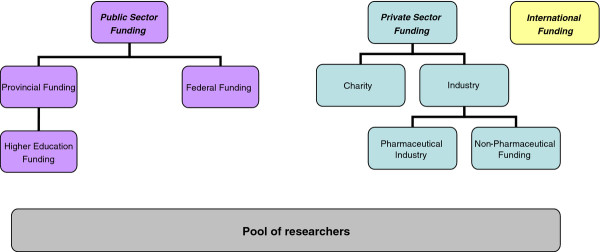
Funding structure of the Canadian health research system.

Broadly speaking, the Canadian health research funding system can be divided into three groups: public sector funding (federal and provincial governments), private sector funding (not-for profit organizations such as charities and foundations, and for profit organizations – industry broadly speaking) and international funding (see Figure 1) [7]; in this analysis, we focus on public sector funding and private not-for profit sector (charities and foundations) funding only.

### Funding organizations in Canada

To obtain data on expenditures of public/charitable-funded research, we compiled a list of the major national granting agencies in Canada, such as the Canadian Institute of Health Research (CIHR), the Natural Sciences and Engineering Research Council (NSERC) and the Social Sciences and Humanities Research Council (SSHRC); the major foundations, such as the Heart & Stroke Foundation of Canada (H&SF of Canada), the Canada Foundation for Innovation (CFI), Genome Canada and the Canadian Health Services Research Foundation (CHSRF); and other major provincial organizations, such as the Fonds de la recherche en santé du Québec (FRSQ). This list was our stating point.

Next, we undertook an internet search for additional organizations. This was achieved by searching links on the websites of organizations already identified (see list mentioned beforehand) and by general internet searches. Next, we used a snowball sampling approach when contacting each funder we found, asking if they were aware of any additional sources of health research funding that we had not captured in our first search [8]. Finally, we made use of the Reference Lists of health research in Canada (which include data on grants and awards collected by the former MRCC and supplied to us by the CIHR) [9] to provide names of organizations that were not captured in our search, either because they have ceased to exist or have changed their name over the years.

Once a list was compiled, we contacted every organization to obtain cardiovascular disease research-related expenditures from 1975 to 2005. Information was provided either directly by the organization or through its annual reports; this occurred in 73% of the cases. When this information was not available or not provided, we made use of the reference lists. These data are not openly available; permission to use the data was obtained from the CIHR or the individual organization. In most cases, the expenditure data reported in the annual reports were also included in the Reference Lists.

### Reference lists of health research in Canada

The Reference Lists of health research contains lists of the extramural research projects (grants and awards) in Canada supported by federal, provincial, and voluntary agencies [9]. The Reference Lists are not a complete record for the grants and awards for a given fiscal year [9]. For the majority of the agencies it includes only those approved prior to July 1 of that year [9]. While some agencies have completed their awards by that date, others such as the MRCC, continue to make awards during the balance of the fiscal year. Other agencies, for valid reasons prefer not to publicize their awards and no record can therefore be included [9]. However, in spite of the fact that the records are incomplete, it reflects the greater part of awards provided for that year.

The Reference Lists do not include agencies outside of Canada, such as the US NIH; Canadian and foreign business enterprises, including the pharmaceutical industry; a number of small, mainly local or regional agencies and foundations; and endowments or other funds at the discretion of universities, hospitals, and affiliated institutions [9].

In particular, for each research project listed, the following information was available:

1. List number used for identifying the project in the indices

2. Name of the recipient of the grant/award

3. Department/faculty and the university/institution where each investigator holds his or her main academic appointment or is located or where the research training is being pursued

4. Title of the research project

5. Project number assigned by the granting agency

6. Year in which the project was initiated

7. Classification by department in which the work is carried out as defined in the Index

8. Funding provided by the agency for the grant or award in the agency’s latest fiscal period and in the previous one if applicable.

### Coding methodology

Previous research has classified funding by field using grant abstracts [10,11] and other grant information [12-14], information on the mission of the funding agency or institute [15] or numbers reported to legislators [16]. The NIH currently uses the Research, Condition, and Disease Categorization (RCDC) coding, which is based on coding information from full text of grant applications. The RCDC method was implemented in 2008 by the NIH at the request of Congress to provide better consistency and transparency in the reporting of its funded research. This method uses sophisticated text data mining (categorizing and clustering using words and multiword phrases) in conjunction with NIH-wide definitions used to match projects to categories, while improving consistency and eliminating variability in the definition of the research categories reported.

For our analysis, we classified CVD funding by examining grant and fellowships/scholarship titles and abstracts (and summary/key words, when available) as we did not have access to the full text of the grant. In particular, two independent reviewers scanned all grant and fellowship/scholarship titles of all research projects funded to assess whether these were CVD-related and eligible for inclusion in our analysis. The grants and fellowships/scholarships were selected if the clinical conditions studied included heart and peripheral vascular diseases, namely acute myocardial infarction, acute coronary syndrome, acute angina and cardiovascular heart disease, heart failure, hypertension or hyperlipidemia. These are the same outcomes examined in the Ontario IMPACT model [17] and were chosen for the sake of comparability with previous work on the topic [18]. The list of grants and fellowships/scholarships of each reviewer were then compared to see if they were consistent with one another; when required, the reviewers met to discuss any inconsistencies regarding a grant/fellowship/scholarship’s inclusion and to reach consensus. This selection method was applied to all organizations and years of our analysis.

Fortunately, we did not have many cases of missing data; we employed linear interpolations in two cases only (one data point for the H&SF of Canada^a^ and the FRSQ,^b^ respectively) to obtain missing expenditure estimates. Furthermore, we generally made less assumptions compared to previous work on the same topic [18]. For example, we did not assume all expenditure data from the H&SF of Canada were relevant to our analysis; we reviewed each individual grant title to assess its inclusion in our analysis.

We estimated the value of expenditures in constant dollars, adjusted by the Consumer Price Index to 2005 dollars [19]. “Constant dollars” refers to the monetary value of prior expenditures expressed in currency units for a fixed, base year (in our case, 2005), after adjusting for the effect of inflation.

## Results

**Figure 2:**
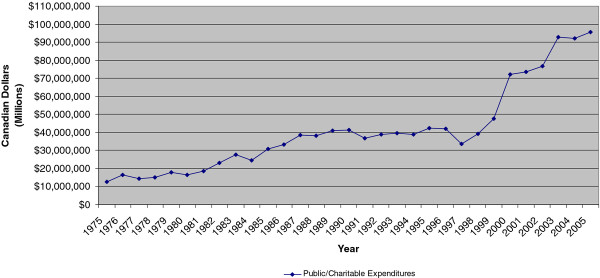
Expenditures on cardiovascular R&D for public/charitable organizations in Canada, 1975–2005.

Table 1 lists the public/charitable organizations found in our search; all organizations, except the Newfoundland & Labrador Centre for Applied Health Research, provided funding for cardiovascular-related research during our analysis period. Figure 2 provides an overview of the trend for cardiovascular-related research for non-profit funding sources in Canada from 1975 to 2005. In 1975, these expenditures were $13 million while in 2005 this value had increased to $96 million (in constant dollars). There is the possibility that we did not capture all organizations that funded CVD research during our analysis period; however, given that most federal, provincial, and voluntary agencies reported to the MRCC [9], we feel that this is not too problematic.

**Table 1:** Cardiovascular disease research funding organizations included in our analysis

**Federal Funding**	
***Granting Agencies***	
·Medical Research Council of Canada (MRCC)/Canadian Institutes of Health Research (CIHR)	
·Canada Council/Social Sciences and Humanities Research Council (SSHRC)	
·Natural Sciences and Engineering Research Council (NSERC)	
·Government of Canada, Indirect Costs Research Program	
***Foundations***	
·Canada Foundation for Innovation (CFI)	
·Canadian Health Services Research Foundation (CHSRF)	
·Genome Canada	
**Provincial Funding**	
***Other Public Organizations***	
·Prince Edward Island Health Research Institute	
·Newfoundland & Labrador Centre for Applied Health Research	
**Charitable Organizations and Foundations**	
***Charitable Organizations***	
·Heart & Stroke Foundation of Canada (H&SF in Canada)	
·British Columbia Health Care Research Foundation/Michael Smith Foundation	
·Alberta Heritage Foundation for Medical Research (Alberta Innovates - Health Solutions since 2010)	
·Saskatchewan Health Research Board/ Health Services Utilization and Research Commission/ Saskatchewan Health Research Foundation	
·Conseil de la recherche en santé du Québec (CRSQ)/Fonds de la recherche en santé du Québec (FRSQ)	
***Foundations***	
·Manitoba Medical Service Foundation	
·Manitoba Health Research Council	
·Banting Research Foundation	
·J.P. Bickell Foundation	
·Nova Scotia Health Research Foundation	
·Dalhousie Medical Research Foundation	
·The Physicians' Services Incorporated Foundation	

**Figure 3:**
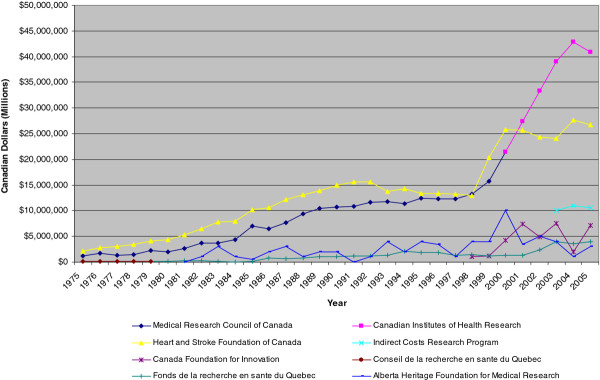
Expenditures on cardiovascular R&D for the top public/charitable organizations in Canada, 1975–2005.

Some organizations have contributed quite a bit to the overall amount of cardiovascular research-related expenditures while others have contributed very little or not at all. For example, in 1975, the H&SF of Canada was the largest funder of CVD research, funding close to 62% of all grants and scholarships/fellowships; the MRCC funded roughly 35% of those research endeavours. These two organizations alone covered the vast majority of CVD-related research initiatives – close to 97% (see Figure 3). At the provincial level, the Conseil de la recherché en santé du Quebec (CRSQ) stood out as one of the top funders (3.2%).

Ten years later, in 1995, the funding landscape in Canada had changed quite a bit – while the H&SF of Canada and the MRCC continued to be the main funders, the proportion of how much they funded had changed a bit. While the MRCC’s importance increased slightly, now funding 38%, the H&SF of Canada proportion of funding had fallen to 41%. Funders at the provincial level were starting to have a larger role in funding, with the Alberta Heritage Foundation for Medical Research (AHFMR) providing funding for close to 16% of all CVD-related grants and the Fonds de la recherche en santé du Quebec (FRSQ) (former CRSQ) funding close to 6%.

In 2005, the CIHR (former MRCC) was the largest funder (42%), followed by the H&SF of Canada (28%), the newly created Indirect Costs Research Program (ICRP) (11%) and the CFI (7%). To undertake research-related activities, institutions need to invest in research infrastructure, such as state-of-the-art equipment, buildings, laboratories, and databases required to conduct research. The CFI was created by the Government of Canada in 1997 as an independent corporation to support research infrastructure. It usually funds up to 40% of a project’s infrastructure costs. The ICRP was created in 2003; through this program, the federal government funds specific research projects each year via its three granting agencies (CIHR, NSERC and SSHRC). The creation of these last two organizations explains, in part, the sharp increase and high expenditure levels observed in the later period of our analysis. At the provincial level the AHFMR and the FRSQ were still major funders, though their importance has decreased a bit (3% and 4%, respectively).

## Discussion

Canadian funding for health research has varied over the last few decades. Our work focused specifically on the magnitude of public/charitable funding on cardiovascular disease (CVD)-related research from 1975 to 2005. These sectors’ spending have increased over this 20-year period but mostly in the last decade. In particular, public/charitable sectors expenditures first rose after 1998, from $39 million to $48 million, and then again from 2000 onwards, from $72 million to just over $90 million.

A closer look at the composition of the public/charitable sectors expenditure trends over time show that the H&SF of Canada and the MRCC have been the main funders and drivers of CVD research in Canada during our analysis period (Figure 3). From 1975 to the mid-80s, the H&SF of Canada and the MRCC funded the majority of CVD-related research. After that, provincial agencies, such as the AHFMR and the FRSQ, started to gain importance. The ICRP and CFI became major funders in the later years of our analysis, after their creation.

This first major increase in CVD funding can be attributed to several factors. In 2000, the MRCC transitioned to the CIHR with a broader mandate and endowed with a larger budget [5]. In addition, after 1999 the H&SF of Canada started funding research chairs in cardiovascular and stroke research, providing additional funding for researchers [9]. Finally, the creation of both the CFI and the CHSRF in 1997 contributed to an increase in overall funding. The second major increase in funding can be explained, in part, by the creation of several other organizations. Genome Canada was established in 2000 to develop and implement a national strategy to support large-scale genomics and proteomics research. The ICRP followed in 2003.

Over our analysis period, public/charitable sectors expenditures increased by a factor of almost 7.5. However, compared to the UK, Canada has spent far less on total public/charitable cardiovascular-related research over the last 30 years. In particular for the public/charitable sectors, Canada spent almost $13 million on cardiovascular-related research in 1975 for a population of about 23 million – roughly $0.60 per person. The UK spent about $4.50 per person, 8 times the Canadian value for a population twice as large [18]. In 1992, seventeen years later, Canada spent about $1.42 per person on cardiovascular-related research, while the equivalent UK value was $4.23. The UK has traditionally spent more on research-related activities than Canada [18], which largely explains this difference. Furthermore, this result may be due to different approaches in estimating expenditure data. Nonetheless, the gap in research expenditures between the two countries has decreased in the last years, attributed largely to creation of the CIHR and other research-funding organizations.

Other research reports that overall biomedical and health services research spending per capita in Canada in 1985 ($17) was slightly more than half that of the US ($30) [20]. More recent data also show that Canada spends substantially less on CVD-related research than the US. Data from the NIH (using the RCDC method) shows that for 2008 about $4.6 billion dollars (USD) were spent on CVD-related research (this includes atherosclerosis, cardiovascular, heart disease, heart disease – coronary heart disease, hypertension and stroke) [21]. (Unfortunately, we do not have the figures for 2005; however, it is likely that this value has not changed much.)

By international standards, the evidence suggests Canada spends less on health-related research than the UK and the US, at least in terms of absolute values; however, to fully gauge the impact of Canada’s research investment would require understanding how CVD research expenditures lead to changes in health. From 1994 to 2004, we witnessed a drop in the adult CVD-attributable death rate of about 30% [22]. The extent to which this is attributable to new research is unclear and beyond the scope of this analysis; these health gains are likely a result of not only research undertaken in Canada but also research undertaken elsewhere. However, new discoveries have undoubtedly played some role. An important area for further research is to quantify the relationship between expenditures and health gains, through a formal return on investment analysis [23]. Other jurisdictions, such as the UK, have undertaken such analyses and found that for every $1 spent on public/charitable CVD-related research, UK citizens would receive an income stream of about £0.09 per year in perpetuity. While this type of analysis would allow us to ascertain whether Canada is spending too much or not on research, this too is beyond the scope of this paper.

These estimates provide policy makers and the CEOs of granting agencies a depiction of funding trends in CVD research from not-for profit organizations; that is, it sheds some light on how public/charitable money has been spent on CVD-related research and where the funds have come from. There is an ongoing debate among policy makers regarding the government’s role in producing scientific and technical knowledge. One of the main issues concerns how much public money should be spent on scientific research and which areas of research should receive funding [13]. These figures can serve as important inputs in planning future research budgets and setting priorities for resource allocation in CVD research. Factors such as public health needs, scientific opportunities, the quality of research proposals, and the maintenance of staffing and infrastructure all need to be taken into account when deciding how to allocate funds [24]. Efforts to plan disease-oriented research activities are also influenced by projections of future patterns of disease and the effects of demographic changes (such as aging) and personal habits (such as tobacco use) [24].

Several limitations of our analysis merit discussion. We attempted to estimate all cardiovascular research-related expenditures for public/charitable sectors. Although we may not have accounted for all expenditures, we captured most of them. More importantly, for most organizations we were able to account for all cardiovascular research-related activities, which enhanced the precision of our analysis. Our analysis also required making some assumptions in a few instances (such as linear interpolation) to reach our final estimates.

Notwithstanding these concerns, we believe that we have reached plausible estimates that not only improve our understanding of this topic but also provide substantial improvements over previous work. Buxton et al.’s time series of estimated public/charitable UK expenditures on medical research in cardiovascular disease was pieced together from a variety of sources and incorporated multiple assumptions and interpolations [18]. We believe that our estimates are more reliable for several reasons. First, we included a review of all grants, awards, fellowships and scholarships. Second, since the majority of our data were from the same source,^c^ we can assume that the data used in our analysis have been reported consistently over time. Third, we employed few linear interpolations in our analysis. Finally, we also made fewer assumptions [18]. Thus, this study’s contributions to the field are two-fold: a detailed description of the health care funding landscape for not-for profit organizations in Canada and a consistent time series of public and charitable sectors expenditure estimates for Canadian CVD-related medical research from 1975 to 2005.

## Conclusion

Public/charitable-funded research expenditures devoted to CVD have increased substantially over the last three decades. By international standards, the evidence suggests Canada spends less on health-related research than the UK and the US, at least in absolute terms. However, this may not be too problematic as Canada is likely to free-ride from research undertaken elsewhere. Understanding these past trends in research funding may provide decision makers with important information for planning future research efforts.

Future work in this area should include the use of these methods and others (such as the RCDC method) to obtain estimates of funded research for other diseases. For example, the NIH provides biennially estimates of the annual support level for various research, condition, and disease categories based on grants, contracts, and other funding mechanisms. It would be interesting to undertake the same exercise for research funded by the CIHR or for all major Canadian funding agencies. These numbers would then enable one to assess whether too little/much was being spent on research for a particular disease given its burden [16]. Furthermore, it may be worthwhile developing a comprehensive approach to determining the health-related and broader social benefits associated with investments in medical research.

## Endnotes

^a^ Only one value (the fiscal year of 1999/00) was interpolated using a linear function as the data were not available (there was no external report available).

^b^ Only one value (the fiscal year of 1983/84) was interpolated using a linear function as the grant titles were not available.

^c^ Data were obtained from either the Reference Lists or from the actual organization (annual reports); expenditure trends for some organizations were from both sources.

## Competing interests

All authors declare they have no competing interests.

## Authors’ contributions

CdO collected, analyzed and interpreted the data, contributed to the conceptualization and design of the analysis and wrote the manuscript. All other authors (VHN, HW, WW, GW, PL and MK) provided their input by interpreting the findings and contributing to the conceptualization and design of the analysis. All authors of this paper have read and approved the final version submitted.

## Funding

This project was funded by the Canadian Institutes of Health Research.

## Pre-publication history

The pre-publication history for this paper can be accessed here:

http://www.biomedcentral.com/1472-6963/12/281/prepub
